# Nationwide surveillance algorithms for tuberculosis among immigrant workers from highly endemic countries following pre-entry screening in Taiwan

**DOI:** 10.1186/s12889-018-6029-x

**Published:** 2018-10-03

**Authors:** Mei-Mei Kuan

**Affiliations:** The Executive Office, Centers for Disease Control, Ministry of Health and Welfare, Taipei, Taiwan

**Keywords:** Pulmonary tuberculosis, Immigrant worker, Post-entry screening

## Abstract

**Background:**

This cross-sectional study was retrospectively performed to assess the trend of tuberculosis (TB) among Taiwan’s immigrant workers from highly TB-endemic countries under an intervention of conducting a 4-round follow-up (at 0–3 days and 6, 18, and 30 months post-migration) screening program with initial chest X-ray (CXR) following an overseas, pre-entry normal CXR.

**Methods:**

The immigrant workers with TB disease enrolled in the Taiwan TB registry database in 2011–2014 were analyzed and linked to an immigrant worker physical exam database to stratify TB case categories of actively screened or not for comparison.

**Results:**

Following pre-entry screening for the admission of CXR-normal immigrant workers from highly endemic countries, the overall TB incidence of 70.6–128.6/100,000 person-years resulted either from a subsequent series of 4-round post-entry active screenings or misalliance algorithms, including passive diagnostics for the illness. Overall, the TB relative risk based on incidence in the immigrant working population was 2.2- to 5.5-fold greater than that among corresponding age Taiwanese, with 14.3% (15.5/100,000 person-years) sputum-smear-positive pulmonary TB (SS+ PTB), 74.2% (80.8/100,000 person-years) sputum-smear-negative (SS-) PTB, and 7.8% (8.5/100,000 person-years) only extra-pulmonary TB (EPTB). Regarding the clinical characteristics, 55.5% TB cases – identified through passive illness diagnostics vs. 44.5% TB cases actively identified through mandatory screenings, were higher in SS+ PTB (adjusted odds Ratio (aOR): 1.5, 95% CI: 1.1–2.0, *P* = 0.008), higher in SC+ PTB (aOR: 1.4, 95% CI: 1.1–1.7, *P* = 0.004), higher in concurrent extra-pulmonary TB (aOR: 8.9, CI: 4.5–7.4, *P* < 0.001), and higher in normal CXR TB (aOR: > 100, CI: 0- > 100, *P* = 0.908). The TB yields of 3rd- to 4th-round screenings were higher than those of 1st- and 2nd-round screenings and ranged from 52.6–65.3 cases per 100,000 screenings in 2013–2014.

**Conclusions:**

The multiple post-entry TB screenings with initial CXR for high-risk immigrants could actively reduce TB transmission by finding SS- TB cases at early stages. The TB yields at post-entry 3rd- to 4th-round screenings might imply a persistent reactivation of latent TB. Adding more sensitive, economical screenings and preventive treatments for latent TB infection is a comprehensive approach for accelerating TB elimination.

## Background

Because several industrialized countries have shown that a high proportion of active tuberculosis (TB) cases among immigrants occur in the first 5 years [1–4], Taiwan has implemented a TB screening program for immigrants from highly endemic countries. Taiwan has been identified as an intermediate TB burden country; in 2011–2014, the TB prevalence was approximately 48–55 per 100,000 people [5]. Of these TB patients, over 70% were aged 50 years or older. Taiwan’s immigrant TB control policy primarily aims to screen active TB cases through chest radiographs. The criteria for admission to Taiwan or to remain in Taiwan for a 3-year residency for new immigrant workers with high TB risk is an overseas, within 3 months beforehand, pre-entry normal chest X-ray (CXR) result. Subsequently, over a 2.5-year follow-up period, there is post-entry screening within 3 days of entry and in the 6th, 18th, and 30th months. However, Taiwan’s TB screening policy for immigrants was updated in January 2014. Since then, the policy has provided TB treatment for immigrant workers with TB and has conducted repatriation only in cases of multi-drug-resistant TB (MDR-TB). Because the epidemiologic data were limited, this study aimed to analyze and assess the TB impact among these entrants and the screening effectiveness from 2011 to 2014. This study aimed to assess the TB burden among workers born outside Taiwan following pre-entry screening by analyzing nationwide data obtained from the TB registry database based on a National Surveillance Network of Communicable Disease (NSNCD) of Taiwan’s Centers for Disease Control (TCDC). Additionally, the effectiveness of Taiwan’s immigrant TB screening policy was assessed through data analysis. Data were derived from the TB registry database linked to the screening program’s immigrant worker physical exam database for obtaining annotation data about cases’ screening round records in cases that were actively identified by TB screening; otherwise, illness was identified passively.

## Methods

A retrospective, cross-sectional study of TB surveillance among immigrant workers based on 4-year (2011–2014) data was conducted. TB data in Taiwan was obtained from the following sources: (1) the national TB registry database based on clinical characteristics information, which is updated by clinicians nationwide; (2) the national database of immigrant workers’ physical examinations with individual health screening dates/CXR outcomes, which is updated by local health divisions; and (3) linkage of (1) and (2) via individual passport numbers to stratify the TB case results by active post-entry screenings or passive surveillance, i.e., illness diagnostics.

### Definition of immigrant workers with TB

According to Taiwan’s policy, newly arrived immigrant workers from highly endemic countries are required to have a verified normal CXR performed overseas (i.e., pre-entry) during pre-entry 3 months and follow-up, i.e., post-entry screenings at 0–3 days (1st round), 6 months (2nd round), 18 months (3rd round), and 30 months (4th round) to obtain residency for a maximum of 3 years. Abnormal CXR cases require subsequent laboratory confirmation of TB and are classified as 1) sputum smear-positive pulmonary cases; 2) sputum smear-negative pulmonary cases, for which the diagnostic criteria include at least two AFB-negative sputum smear examinations, the decision by a clinician to treat with a full course of anti-TB chemotherapy, or a sputum culture-positive but AFB-negative sputum examination; or 3) extra-pulmonary TB cases, in which the patient exhibits TB purely in organs other than the lungs. A patient in whom both pulmonary and extra-pulmonary TB has been diagnosed is classified as a pulmonary case i.e., concurrent extra-pulmonary TB.

### Statistical analysis

In total, data from 2080 TB-positive immigrant workers were obtained from the TB registry database and were linked with the National Immigrants Health Examination System database to determine their post-entry screening round or the illness identified. The individuals’ sex and clinical characteristics, including negative, positive, or unknown/not performed CXR results, sputum smear results, sputum culture results, and TB type (pulmonary TB: pulmonary TB lesions exist; extra-pulmonary TB-) were obtained from the TB registry database i.e., the National TB Database.

The TB impact during the study period was calculated using the following formula: the TB incidence rate was calculated as the number of TB cases newly detected at 0–36 months post-entry following pre-entry normal CXR results during the study period divided by the population of immigrant workers or Taiwanese citizens per year. The populations of Taiwanese citizens and immigrant workers were obtained from the Ministry of the Interior and the Ministry of Labor, respectively [6]. The sex- and age (20–49)-specific TB incidence rates were calculated as the number of TB cases aged 20–49 divided by the number of corresponding population, i.e., sex- but not age-stratified immigrant workers, due to the limited population databases available from the Ministry of Labor; however, because more than 98% of the immigrant workers were aged 20–49, the bias of under-estimation due to a denominator without age-stratification could almost be neglected.

The strength of the associations between the pairs of variables (i.e., incidence rate detected in Taiwan for each nationality vs. overall original incidence rates estimated by the WHO [7]) was calculated using Spearman rank order correlations. The relative risk of TB was calculated as the TB incidence rate for the target group (e.g., immigrant workers aged 20–49) divided by the TB incidence rate of the reference group. For example, the incidence rates of the reference groups (i.e., TB rates among Taiwanese females and males aged 20–49) were 25.0/100,000 [5] and 32.0/100,000 person-years [5], respectively, during 2011–2014.

Because the majority of TB patients were identified actively through health screening efforts or passively through illness diagnostics, we divided the studied TB case population into two categories: active surveillance algorithms (case finding via active screening) vs. identified via passive surveillance algorithms (cases finding via illness diagnostics or misalliance reasons). Category comparisons were analyzed by the Pearson’s chi-squared test for assessment of the difference between the independent variables. Furthermore, a logistic regression was employed to assess the impact of multiple variables on the binary surveillance algorithms and to adjust for the effects of other variables by using Epi Info version 7 (Centers for Disease Control and Prevention, Atlanta, GA, USA). The outcomes of two runs of multiple logistic regression analyses for risk factors (independent variables) of passive surveillance algorithm (dependent variable) vs. active surveillance algorithm (dependent variable) or vice versa were respectively presented.

### Yield of post-entry screening

The screening yield was defined as the proportion of the number of TB cases or sputum-smear-postive TB cases detected per 100,000 individuals screened (i.e., screenings). When calculating the yield of TB screenings for assessing effectiveness, the number of TB cases (numerator) was obtained from the Taiwan national TB registry database matched with the immigrant worker physical exam database. The number of individuals screened, for the denominator, was directly obtained from the physical exam database. Comparisons of yields utilized t-tests based on 2-tail analysis, and *P* < 0.05 was deemed as significant following the F test.

#### Ethics statement

This study was approved by the Institutional Review Board of the Taiwanese CDC under identification no. TwCDCIRB104104.

## Results

### Immigrant workers with TB and the relative risk of TB

**Fig. 1:**
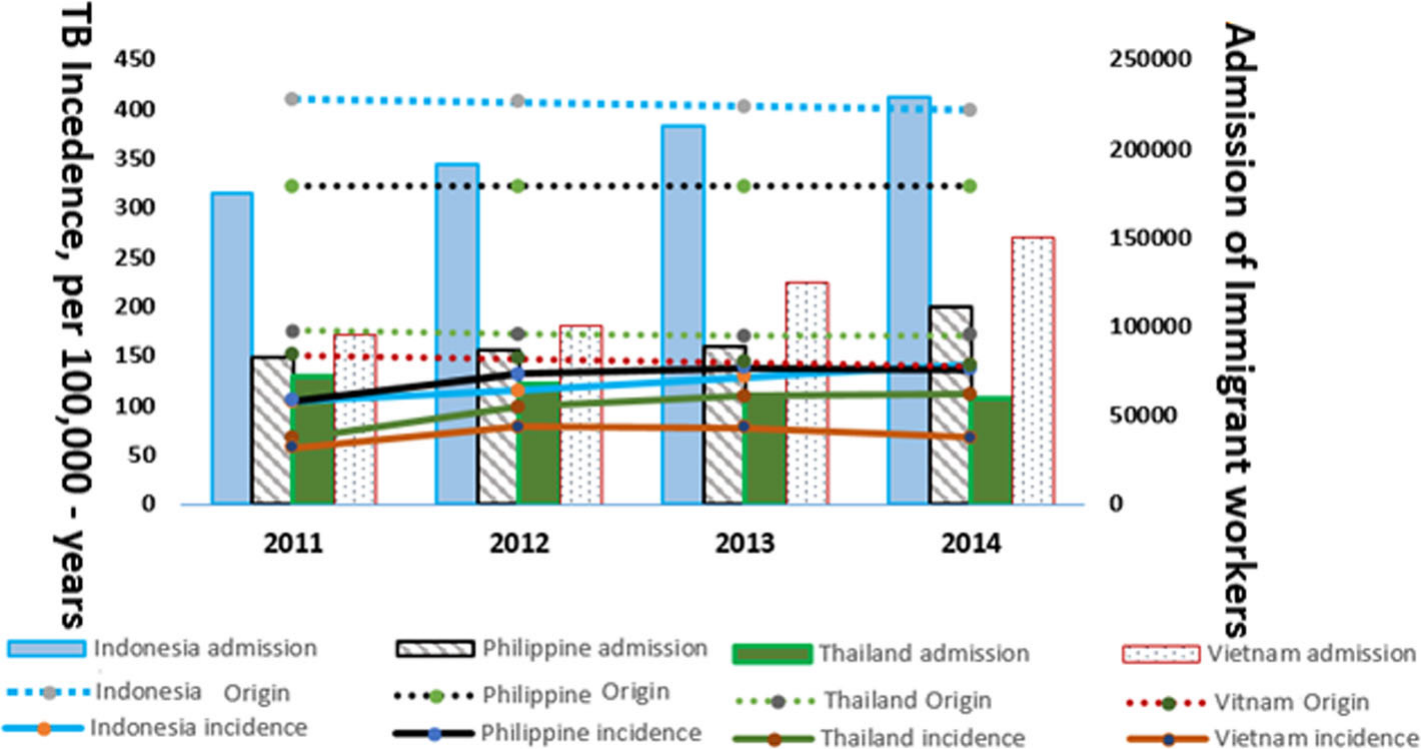
Annual trends of TB incidence rates among immigrant workers (presented as curve charts) or admission numbers of immigrant workers (presented as bar charts) in Taiwan during 2011–2014. There was an increasing trend in TB in Indonesia (TB incidence rates: 105.7–135.3/100,000 person-years) and a parallel increasing trend in the admission numbers of Indonesian immigrants (151,588-213,486). With (2011–2013) or without (2014) a relocation policy for TB cases, immigrant workers from highly endemic countries showed no significant differences in TB incidence between 2013 and 2014, with the exception of a decline in Vietnam and an increase in Indonesia. In parallel, the fluctuations of the TB incidence rates among immigrant workers of each nationality in Taiwan were significantly positively associated with (*R* = 0.74, 2011–2014) and lower than those in the original countries

According to the Taiwan national TB registry database during 2011–2014, a total of 2080 cases of TB of all types (377, 481, 566, and 656 cases annually) were newly identified among the average annual 477,992 (389249–566,735) immigrant workers [6] who originated from southeastern Asia following pre-entry screenings with normal CXR results (Table 1, Fig. 1). The TB incidence rates for these entrant workers from the top four highly TB-endemic countries (Indonesia, the Philippines, Thailand and Vietnam) were 122.9, 113.3, 89.6, and 64.3 TB cases/100,000 person-years, respectively (Table 1, Fig. 1). The sex- and age (20–49)-specific TB incidence rates for females (62.7–132.2/100,000 person-years) and males (65.4–164.0/100,000 person-years) among immigrant workers were compared with those of Taiwanese females (25/100,000 person-years [5]) and Taiwanese males (32/100,000 person-years [5]), resulting in relative risks (RRs) of 2.5–5.3 among female immigrant workers and 2.0–5.1 among male immigrant workers (Table 1, Fig. 1). Moreover, the TB incidence rates estimated herein among immigrant workers in Taiwan were much lower than but positively associated with those of the WHO-estimated TB incidence rates in their countries of origin [7] (i.e., *R* = 0.74 from the Spearman rank order correlation, according to the immigrant incidences estimated herein vs. WHO estimates [7] in 2011–2014) (Fig. 1).

**Table 1:** The TB incidence among immigrant workers in Taiwan, 2011–2014

Characteristics	Total	Vietnam	Indonesia	Philippines	Thailand	Miscellaneous^e^
All types of TB^a^	2080	333 (16.0%)	1001 (48.1%)	476 (22.9%)	250 (12.0%)	20 (0.7%)
Female	1246 (59.9%)	139	7400	321	34	12
Male	834 (40.1%)	194	261	155	216	8
Age < 20	12 (0.6%)	10	2	0	0	0
Age 20–49 (female)	2046 (98.3%)	318	999	471	242	16
Age > 49	22 (1.0%)	5	0	5	8	4
Average annual female immigrant worker population	283,291	48,633	165,575	58,425	10,657	–
Average annual male immigrant worker population	194,700	69,239	36,740	34,121	54,597	–
TB incidence for all immigrant workers^b^, 10–5/y		70.6	123.6	128.6	95.8	
TB incidence for female immigrant workers^b^, 10–5/y		71.5	111.7	137.4	79.8	
TB incidence for male immigrant workers^b^, 10–5/y		70.0	177.0	113.6	98.9	
TB RR^c,d^ female immigrant workers vs. female Taiwanese		2.9	4.5	5.5	3.8	
TB RR^c,d^ of male immigrant workers vs. male Taiwanese		2.2	5.5	3.6	3.1	

Values are numbers, except as indicated

^a^ All types of TB, including 1) smear-positive pulmonary cases, 2) smear-negative pulmonary cases, and 3) extra-pulmonary cases.

^b^ TB incidence for female/male immigrant workers: TB cases at 0–30 months post-entry/number of immigrants.

^c^ TB risk for Taiwanese females: 25 per 10^5^-y in 2011–2014; TB risk for Taiwanese males: 32 per 10^5^-y in 2011–2014.

^d^ TB RR: TB risk ratio (or TB relative risk) for calculating the respective TB risks of female or male immigrant workers vs. those of female or male Taiwanese

^e^ Miscellaneous: Malaysia (0.1%), Mongolia (0.1%), and countries of origin not specified (0.7%).

### Clinical characteristics according to diagnostics

**Fig. 2:**
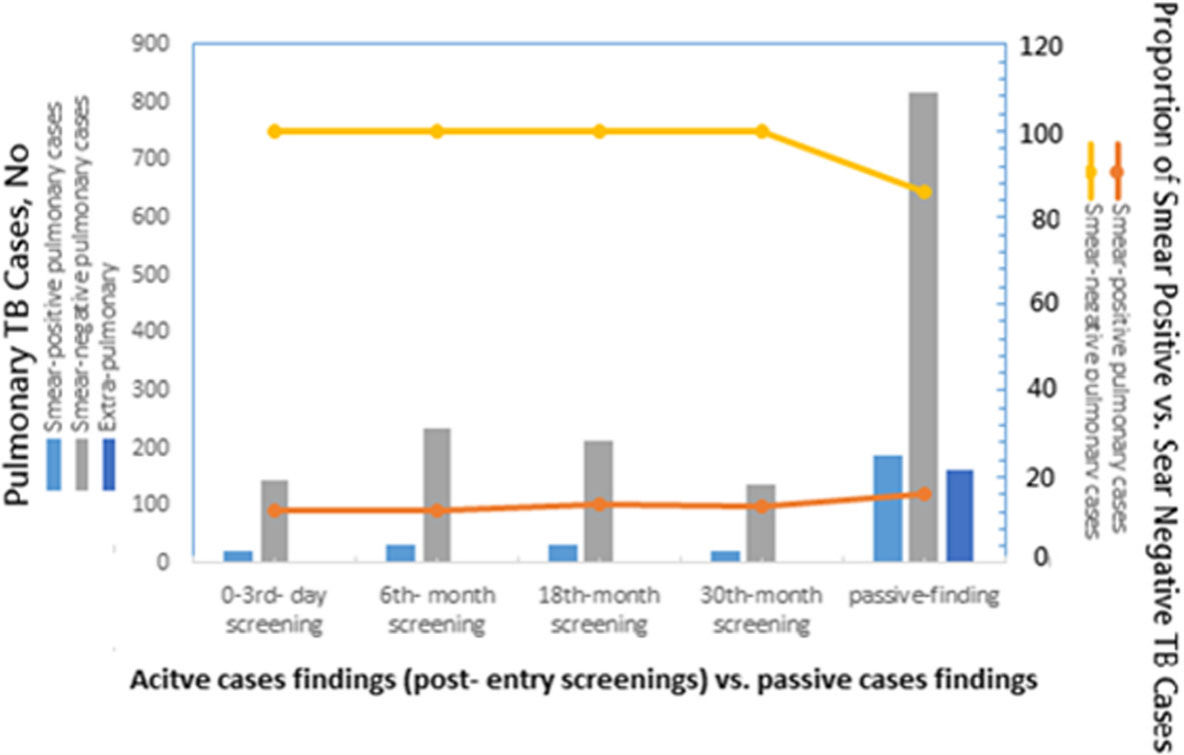
Among immigrant workers with TB whom passively identified through the misalliance surveillance algorithm including illness diagnostics compared to actively identified through mandatory screenings surveillance algorithm with a higher clinical characteristics proportion of 16.4% (189/1154) vs. 11.7% (108/926) of the smear-positive pulmonary TB cases and a lesser proportion of 70.8% (817/1154) vs. 78.5% (727/926) of the smear-negative pulmonary TB cases i.e., at higher proportions in SS+ PTB (aOR: 1.5, 95% CI: 1.1–2.0). The TB yields of the 0-, 6-, 18-, and 30-month post-entry screenings of these immigrant workers were 24.4 (95%CI 12.5–36.3), 40 (95%CI 25.1–54.9), 52.6 (95%CI 25.1–80), and 47.4 (95%CI 31.5–63.3) cases per 100,000 screenings, respectively; the respective detection proportions were 7.4% (153/2080), 13.9% (289/2080), 13.9% (290/2080), and 8.6% (179/2080) in Taiwan

Overall, all TB cases were classified as 14.3% (297/2080; 15.5/100,000 person-years) smear-positive pulmonary TB of high infectivity, 74.2% (1544/2080; 80.8/100,000 person-years) smear-negative pulmonary TB of less infectivity, and 7.8% (164/2080; 8.5/100,000 person-years) extra-pulmonary TB (Table 1-3). The sputum culture-positive abundance was approximately 41.9%, with 0.5% MDR-TB (Table 2, Fig. 2).

**Table 2:** Clinical characteristics of 2080 immigrant workers with TB identified via passive versus active surveillance algorithms during 2011–2014

Variables	Total *N* = 2080	No. (%) Passive and unknown^a^ *N* = 1154	No. (%) Active^b^ *N* = 926	*P* value^c^
Sex				0.34
female	1246	703 (60.9)	543 (58.6)	
male	834	473 (56.7)	361 (43.3)	
Age				0.59
< =29	1149	644 (60.1)	505 (47.9)	
> =30	931	510 (41.2)	421 (30.1)	
Nationality				0.034^d^
Indonesia	1001	562 (48.7)	439 (47.4)	
Mongolia	2	2 (0.4)	0 (0)	
Thailand	250	121 (10.5)	129 (13.9)	
Malaysia	2	2 (0.2)	0 (0)	
Philippine	476	252 (21.8)	224 (24.2)	
Vietnam	333	199 (17.2)	134 (14.5)	
Chest X-ray radiography				< 0.00001^d^
Normal	217	217 (18.8)	0	
Abnormal non-cavity	1674	816 (70.7)	859 (92.8)	
Abnormal w/cavity	157	92 (8.0)	65 (7)	
Atypical	19	17 (11))	2 (0.2)	
Microbiology status				< 0.00001^d^
AFB smear-positive	298	185 (16.0)	113 (12.2)	
AFB smear-negative	1576	785 (68.0)	791 (85.4)	
Extra-pulmonary (EP) TB^e^	164	164 (14.1)	0	
Concurrent EPTB^f^	88	78 (6.9)	10 (1.1)	
Sputum Culture				0.055
Positive	871	500 (43.3)	374 (40.4)	
Negative	1124	594 (51.5)	531 (57.3)	
MDR-TB	10	8 (0.7)	2 (0.2)	0.21

^a^ Passive algorithms and unknown: TB cases identified via illness diagnostics, other clinical treatments during hospitalization, or few of them were unenrolled in immigrant worker physical examination systems due to changes in passport numbers

^b^ Active algorithms: TB cases identified via post-entry screening and were enrolled in immigrant worker physical examination systems

^c^ *p* value: Two-sided values were from Pearson’s chi-squared test. TB cases identified via illness diagnostics vs. TB cases identified via post-entry screenings based on each testing variable, e.g., sex, age, microbiology of pulmonary status, CXR, and MDR-TB

^d^ Statistically significant¸ *P* < 0.05

^e^ Extra-pulmonary (EP) TB: having extra-pulmonary TB infection with no lung involvement, i.e., X-ray normal, smear negative, and sputum culture negative

^f^ Concurrent EPTB: having extra-pulmonary TB infection with X-ray abnormal or smear positive or sputum culture positive. Abbreviations: AFB, acid-fast bacilli; MDR, multidrug-resistant to at least isoniazid and rifampin

Database linkage for stratifying active surveillance vs. passive surveillance algorithms including misalliance reasons, and a binary comparison for characteristics analysis.

Linking the 2080 TB-positive immigrant workers in the national registry TB database with the immigrant worker physical exam database revealed that 55.5% (1154/2080) of TB cases were identified through the misalliance algorithm, including passive illness diagnostics, and 44.5% (926/2080) of TB cases were identified via the active algorithm of post-entry screenings (Table 2).

Certain demographic or clinical characteristics were assessed if significant differences by Chi test for binary comparing the surveillance of TB cases via misalliance algorithm (e.g., illness diagnostics and misalliance reasons) vs. active detection (i.e., health screening) (Table 2). The outcomes of the binary assay for clinical characteristics indicated a higher proportion of 16.4% (189/1154) vs. 11.7% (108/926) of smear-positive pulmonary TB cases and a lesser proportion of 70.8% (817/1154) vs. 78.5% (727/926) of smear-negative pulmonary TB cases among passively identified TB cases compared with the actively identified cases (Fig. 2). In other words, it was shown that with the clinical characteristics of 55.5% of TB cases identified through the misalliance algorithm surveillance including illness diagnostics vs. 44.5% TB cases actively identified through mandatory screening surveillance were present at higher proportions in SS+ PTB (aOR: 1.5, 95% CI: 1.1–2.0, *P* = 0.008), higher in SC+ PTB (aOR: 1.4, 95% CI: 1.1–1.7, *P* = 0.004), higher in concurrent extra-pulmonary TB (aOR: 8.9, CI: 4.6–7.4, *P* < 0.001), and higher but not statistically significant in normal CXR TB (aOR: > 100, CI: 0–100, *P* = 0.908) (Fig. 2, Table 2-3).

**Table 3:** Multiple logistic regression analyses for risk factors of passive vs. active TB surveillance algorithm and vice versa were respectively presented

Covariates	Passive surveillance (illness diagnostics)		Active surveillance (post-entry screenings)	
	adjusted OR (95%CI)	*P*-value	adjusted OR (95%CI)	*P*-value
Chest X-ray normality	4,025,604.84 (0.0–1.0E12)	0.909	0.0 (0- > 1.0E12)	0.909
Sputum culture positivity	1.5 (1.2–1.8)	0.0004	0.9 (0.6–0.8)	0.001
Sputum smear positivity	1.5 (1.1–2.0)	0.005	0.7 (0.5–0.9)	0.006
Concurrent TB extrapulmonary TB	8.9 (4.5–17.4)	0.000	0.1 (0.1–0.2)	0.000
Vietnam nationality	1.3 (1.0–1.7)	0.032	0.7 (0.5–9)	0.022

Statistically significant (*P* < 0.05 for 95%)

*OR* = Odds ratio; *CI* =  confidence interval

### Mandatory post-entry screening

The overall TB yield of the multiple follow-up screenings performed 0–30 months after entrance for immigrant workers from highly endemic countries was 40.5 per 100,000 screenings (911/2,248,143; Table 4) in 2011–2014. In terms of the effectiveness of each round of screening, the TB yields of the 0-, 6-, 18-, and 30-month post-entry screenings of these immigrant workers were 24.4 (95%CI 12.5–36.3), 40 (95%CI 25.1–54.9), 52.6 (95%CI 25.1–80), and 47.4 (95%CI 31.5–63.3) cases per 100,000 screenings, respectively; the respective detection proportions were 7.4% (153/2080), 13.9% (289/2080), 13.9% (290/2080), and 8.6% (179/2080) in Taiwan (Fig. 2). The overall yields of sputum-smear-positive PTB or sputum-culture-positive TB at post-entry screening among these immigrant workers were 5.1 and 21.2 per 100,000 individuals (Table 2), respectively, in 2011–2014 (Table 4).

**Table 4:** Yields of TB, sputum smear positive (SS+), and sputum culture positive (SC+) at post-entry screening conducted during the tracking period (0–30 months) for immigrant workers with normal CXR at pre-entry screening

Year	Screened round	Screenings, n	Positive cases, n			Yield, per 10^5^ screenings^a^		
			TB	SS+	SC+	TB^a^	SS + ^a^	SC + ^a^
2011	1 (0–3 day)	149,317	27	3	9	18.1	2.0	6.0
2012	1 (0–3 day)	132,614	31	3	8	23.4	2.3	6.0
2013	1 (0–3 day)	159,541	56	8	21	35.1	5.0	13.2
2014^b^	1 (0–3 day)	187,022	39	4	17	20.9	2.1	9.1
2011	2 (6 month)	160,325	43	4	16	26.8	2.5	10.0
2012	2 (6 month)	169,015	77	11	30	45.6	6.5	17.7
2013	2 (6 month)	165,278	66	8	28	39.9	4.8	16.9
2014^b^	2 (6 month)	216,736	103	12	47	47.5	5.5	21.7
2011	3(18 month)	117,316	37	8	18	31.5	6.8	15.3
2012	3 (18 month)	134,122	61	8	21	45.5	6.0	15.7
2013	3 (18 month)	145,463	97	7	39	66.7^c^	4.8	26.8
2014^b^	3 (18 month)	142,957	95	12	31	66.5^c^	8.4	21.7
2011	4 (30 month)	78,127	29	6	13	37.1	7.7	16.6
2012	4 (30 month)	81,254	33	4	11	40.6	4.9	13.5
2013	4 (30 month)	98,816	54	7	20	54.6^c^	7.1	20.2
2014^b^	4 (30 month)	110,240	63	7	27	57.1^c^	6.3	24.5
Total		2,242,743	911	112	356	40.5	5.1	21.2

^a^ Yield: Cases per 100,000 individuals screened, defined as the number of positive cases detected through screening divided by the number of individuals screened. The total numbers of TB, TB with SS+ cases, and TB with SC+ (numerator) were obtained from the TB registry database matched with the physical exam database. The linked cases (i.e., 22 actively identified TB cases) documented by a screened date of “other” are not shown in this table. The numbers of individuals screened (denominator) were obtained from the physical exam database

^b^ 2014: the year of providing therapy for TB and repatriating only multiple-drug-resistant TB

^c^ Yield of TB larger than cutoff, i.e. > 50 per 100,000 screenings [6]

## Discussion

Immigrant workers with TB likely benefit from early detection via these mandatory multiple screening interventions. The overall TB burden among entrant workers to Taiwan was reduced; most TB cases (74.2%; 80.8/100,000 person-years) were sputum smear-negative and assumed less infectivity in the early pathogenesis stage [8, 9]. Only 14.3% of cases (297/2080; 15.5/100,000 person-years) were sputum smear-positive and of high infectivity [8, 9] during the 3 years post-entry, a lower percentage than those previously reported at pre-entry screenings [3]. However, very few bacilli are sufficient to cause infection [8]. Therefore, we prefer not to neglect or delay therapy for immigrant workers with smear-negative pulmonary TB according to the WHO guidelines, especially those who were hired to provide long-term care for vulnerable people or the elderly. Nonetheless, immigrant workers with normal CXRs from highly TB-endemic Southeast Asian countries sustained a 2.1- to 5.5-fold higher TB incidence rate than domestic residents (i.e., Taiwanese citizens aged 20–49) during their 0–3 years of residency following multiple interventions of pre- and post-screenings. However, among immigrants, the TB prevalence of 70.6–128.6 cases per 100,000/year of each nationality in Taiwan was lower than those of their original countries (i.e. 140–410 cases per 100,000/year [7]). These lower trends were attributed to the screening interventions combined with the TB repatriation policy during the study period in Taiwan. Additionally, in parallel, the fluctuations of the TB incidence rates among immigrant workers of each nationality in Taiwan were significantly positively associated with (R from Spearman rank order = 0.74, 2011–2014) but lower than those in the countries of origin. However, the bias of lacking corresponding age-specific original incidence information for comparison might be due to the following: 1) younger adults are at less risk than the elderly, and immigrant workers are not representative of the total young population in their original countries, 2) immigrant workers are selected from the higher TB-risk groups among the corresponding young population, with a certain characteristic such as a lower socioeconomic status. Moreover, the fluctuations in the annual TB incidence rate among these foreign nations, except for the Philippines, did not exhibit an increasing trend without (2014) vs. with (2011–2013) the TB repatriation policy (Fig. 1).

In terms of screening effectiveness of multiple rounds of post-entry active screening, nearly 2,248,143 post-entry CXR screening episodes were performed that could have detected some (44.5%) of the TB cases earlier and had reduced potential infectiousness. However, screening also missed TB cases (55.5%) that were subsequently detected by passive illness diagnostics with a relatively higher proportion of smear-positive pulmonary TB cases (aOR: 1.5, *P* < 0.05) and higher ORs for normal CXR (i.e., 18% vs. 0) and extra-pulmonary location (i.e., 14.1% vs. 0). Although pure extra-pulmonary TB (EPTB) cases are usually not infectious [2], the condition still brings diagnostic challenges and significant suffering; therefore, healthcare workers need to remain suspicious of immigrants presenting with unexplained symptoms [2]. Moreover, the concurrent extra-pulmonary TB cases in the passively identified group had a higher risk (aOR 8.9: CI 4.5–17.4, *P* < 0.0001). These severe TB patients present with concurrent EPTB, developing a more serious condition that cannot prevent *M. tuberculosis* bacilli from extending beyond the lung parenchyma [10], which has been deemed as contributing to TB transmission [10].

After the overseas screenings within 3 months prior to entrance and accepting a cutoff yield value of 50 cases per 100,000 individuals screened [11], the TB yields of 1st- to 2nd-round follow-up post-entry screenings performed at post-entry 0-days and at 6 months were below the cutoff i.e.,< 50 cases per 100,000 screening [11] and the 2nd-round yields were higher than those of the 1st round (*P* = 0.02). This observation might mean that an overseas screening followed by 2 additional screenings within the first 6 months post-entry results in a TB yield of below the cutoff. Nevertheless, whether the frequency of screening should be adjusted merits further consideration. For example, conducting post-entry screening at 0–3 days could urge immigrant workers to undergo health examinations in their countries of origin prior to entry into Taiwan. The TB yields of the 3rd- and 4th-round screenings performed in the post-entry 18–30-months follow-up period were higher than those of the 1st- and 2nd-round of screenings (*P* = 0.01) and ranged from 52.6–65.3 cases per 100,000 screenings i.e., larger than cutoff (> 50 TB cases per 100,000 screenings) in 2013–2014, encompassing the initial year without mandatory repatriation for foreign workers with TB. Thus, the persistent high TB yields of our follow-up screenings performed at 18–30 months after entrance were different from those reported by Erkens et al. [11], who suggested that the yields of follow-up screenings were low after the 3rd round (i.e., at the 18th month) and suggested that screenings could be limited to a period of 1 year. However, this prolonged, high yield (incidence) trend was in agreement with the findings in studies of low-incidence countries (i.e., a persistent high incidence of TB in immigrants) [4] and might largely be due to the reactivation of latent TB; these individuals could not be successfully screened in advance at pre-entry or in the 1st or 2nd rounds of screening because they may have had latent infections with normal CXR results. Thus, the current multiple screening program based on CXR has limitations, which is consistent with a previous report of a large pre-employment TB screening program based on CXR that produced a low yield in the PTB detection [12]. Therefore, the outcomes of the 2.5 years of follow-up screenings suggest the following. 1) The persistent TB incidence and the fact that approximately half of the patients with PTB among immigrants were passively detected indicate the insufficiency of the mandatory screening premise on CXR performed for mitigating TB. 2) This persistently, relatively high TB incidence and yield for follow-up screening could be improved by an alternative consideration that if it were possible to reduce the pool of latently infected immigrants, the incidence caused by re-activation would be reduced, and follow-up screening could be considered to be largely spared [11] e.g., approximately 2,248,143 post-entry CXRs (Table 4), if pre-entry LTBI detection and treatment are adopted. Moreover, many countries have already tremendously mitigated the TB burden by implementing CXR combined with LTBI screenings [13, 14]. This finding also implies that the effectiveness of screening programs could be improved by incorporating other, more sensitive algorithms [12, 15].

### Limitations

By linking a TB registry database of longitudinal informatics with the immigrant worker health physical examination database of non-longitudinal informatics, the author resolved the gap between the two databases and interpreted the data. However, there are several limitations of this study. 1) There are misalliance reasons, including presumably immigrant workers who are lost to follow-up each year or change their passport numbers, which were beyond the scope of the present study. Based on comparing the population data derived from the Ministry of Labor and from the National Immigration Agency, approximately 50,000 people were lost to follow-up. 2) The TB yield of screenings might be underestimated due to some immigrants who chose to return home who were unwilling to complete the TB diagnostics and were not enrolled as confirmed cases. 3) A limitation scenario of this study is that the Taiwanese population that was not actively screened for TB might cause an underestimation in the TB incidence estimation. 4) The proportions of these immigrant workers with TB who were reactivation or transmission cases were not defined by the lab.

## Conclusions

Overall, a mandatory intervention of admission approval and multiple screenings based on CXR could reduce the TB impact among high-risk immigrants by actively detecting SS- PTB cases with low infectivity at the early pathogenesis stage. The present program of multiple post-entry screenings except for the 1st and 2nd rounds, which had a TB yield below the cutoff, might merit continuation for immigrants from highly endemic countries i.e., TB incidence > 100 per 100,000, following their pre-entry screening. Nevertheless, some PTB cases being detected by a misalliance algorithm and a relatively high TB incidence was sustained in the 3rd and 4th rounds, which might be most attributed to the high risk of reactivation of latent TB in individuals who had no abnormalities on their chest radiographs at the moment of screening. Incorporating more sensitive algorithms, including economic latent TB screenings combined with LTBI treatment e.g., isoniazid preventive therapy, in entry screening for high-risk immigrants might be an effective and comprehensive approach for accelerating TB elimination.

## Abbreviations

CXR: Chest X-ray
EPTB: Extra-pulmonary TB
NSNCD: National Surveillance Network of Communicable Disease
OR: Odds ratio
PTB: Pulmonary TB
SC-: Sputum culture negative
SC+: Sputum culture positive
SS-: Sputum-smear-negative tuberculosis
SS+: Sputum-smear-positive.
TB: Tuberculosis
TCDC: Taiwan Centers for Disease Control

## References

[1] Pareek M, Baussano I, Abubakar I, Dye C, Lalvani A (2012). Evaluation of immigrant tuberculosis screening in industrialized countries. Emerg Infect Dis.

[2] Ködmön C, Zucs P, van der Werf MJ (2016). Migration-related tuberculosis: epidemiology and characteristics of tuberculosis cases originating outside the European Union and European economic area, 2007 to 2013. Euro Surveill.

[3] Aldridge RW, Zenner D, White PJ, Muzyamba MC, Loutet M, Dhavan P (2016). Prevalence of and risk factors for active tuberculosis in migrants screened before entry to the UK: a population-based cross-sectional study. Infect Dis.

[4] Vos AM, Meima A, Verver S, Looman CW, Bos V, Borgdorff MW (2004). High incidence of pulmonary tuberculosis persists a decade after immigration, the Netherlands. Emerg Infect Dis.

[5] Taiwan Centers for Disease Control, Department of Health. Taiwan Tuberculosis Control Report . Available from: https://www.cdc.gov.tw/english/infectionreport.aspx?treeid=3847719104be0678&nowtreeid=ffb51203f16bfe57.

[6] Taiwan Ministry Interior. Monthly Bulletin of Interior Statistics. Available from: https://www.moi.gov.tw/files/site_stuff/321/1/month/month_en.html.

[7] The World Bank. Incidence of tuberculosis (per 100,000 people). Available from: http://data.worldbank.org/indicator/SH.TBS.INCD.

[8] Sepkowitz KA (1996). How contagious is tuberculosis?. Clin Infect Dis.

[9] Kuan MM, Yang HL, Wu HS (2014). Tuberculosis among newly arrived foreign spouses before obtaining citizenship, Taiwan, 2006-2011. Int J Tuberc Lung Dis.

[10] Lin CY, Chen TC, Lu PL, Lai CC, Yang YH, Lin WR (2013). Effects of gender and age on development of concurrent extrapulmonary tuberculosis in patients with pulmonary tuberculosis: a population based study. PLoS One.

[11] Erkens C, Slump E, Kamphorst M, Keizer S, van Gerven PJ, Bwire R (2008). Coverage and yield of entry and follow-up screening for tuberculosis among new immigrants. Eur Respir J.

[12] Eisenberg RL, Pollock NR (2010). Low yield of chest radiography in a large tuberculosis screening program. Radiology.

[13] Muyoyeta M, Maduskar P, Moyo M, Kasese N, Milimo D, Spooner R (2014). The sensitivity and specificity of using a computer aided diagnosis program for automatically scoring chest X-rays of presumptive TB patients compared with Xpert MTB/RIF in Lusaka Zambia. PLoS One.

[14] Pareek M, Greenaway C, Noori T, Munoz J, Zenner D (2016). The impact of migration on tuberculosis epidemiology and control in high-income countries: a review. BMC Med.

[15] Van’t Hoog AH, Onozaki I, Lonnroth K (2014). Choosing algorithms for TB screening: a modelling study to compare yield, predictive value and diagnostic burden. BMC Infect Dis.

